# Modern contraceptive methods knowledge and practice among blind and deaf women in Ethiopia. A cross-sectional survey

**DOI:** 10.1186/s12905-019-0850-y

**Published:** 2019-11-29

**Authors:** Awol Seid Yimer, Lebitsi Maud Modiba

**Affiliations:** 1 Ethiopian Public Health Association, P.O.Box: 7117, Addis Ababa, Ethiopia; 2 Department of Health Studies, University of South Africa, TvW Building 7-160, Pretoria, P.O.Box: 329 South Africa

**Keywords:** Blind and deaf women, Contraceptive methods, Knowledge, Utilization, Ethiopia

## Abstract

**Background:**

Evidences from various parts of the world reveal that women with disabilities are facing widespread barriers in accessing public services. Service providers and program managers do not grasp the relevance of their work and interventions in addressing the sexual and reproductive health needs of women with disabilities. The present study therefore aimed to assess family planning knowledge and practice among women with sensory disabilities.

**Methods:**

A mixed method approach using quantitative and qualitative methods was employed to collect the data. The study included 326 blind and deaf women using respondent driven sampling technique and 29 purposely selected key informants. We carried out the study from August 2016–April 2017. The quantitative data were analyzed using SPSS and the qualitative analysis was done using Open code software version 4.02 and triangulated with the quantitative findings.

**Results:**

The findings showed that nearly two third of the respondents of were sexually active. The majority (97.2%) of study respondents had heard about FP methods, however the level of comprehensive knowledge on modern contraceptive methods was 32.5%. The prevalence of unwanted pregnancy was 67.0% and abortion was 44%.

Almost half of sexually active respondents ever used modern contraceptive methods, yet the contraceptive prevalence at the time of survey was 31.1%. Implants were the most commonly used (51%) contraceptive method among current users.

**Conclusions:**

The use of modern contraceptive methods among women with sensory disabilities was low. Thus, the government and concerned organizations need to address the attitudinal, social, and physical barriers women with sensory disabilities are facing while seeking, accessing to and using family planning services.

## Background

The World Health Organization (WHO) global disability action recognizes disability as a public health, human rights and development priority issue. Disability is a human rights issue since person with disabilities (PWDs) experience inequalities and subject to multiple rights violations including violence, abuse, prejudice, disrespect, and denied autonomy [1]. The UN convention on the rights of persons with disabilities (UNCRPD) marks the paradigm shift by applying a human rights-based approach to disability and claiming an accessible and inclusive society for all [2].

Article 25 of the Convention guarantees persons with disabilities the right to access the same range, quality and standard of free or affordable health care and programs as provided to other persons, including those in sexual and reproductive health (SRH). Article 23 of the Convention points out the rights of PWDs to decide freely and responsibly on the number and spacing of their children and to have access to age-appropriate information, reproductive and family planning services including the means necessary to enable them to exercise these rights. The article further mentions the importance of taking effective actions to eliminate discrimination against PWDs in all matters relating to marriage, family, parenthood and relationships.

WHO/UNFPA, the Program of Action of the International Conference on Population and Development (1994) and the Vienna Declaration (1993) cite these basic reproductive health rights [3–5]. Ethiopia, being a signatory of the above conventions, protocols and needs to ensure that PWDs enjoy these SRH rights. The fourth strategic theme of the national health sector transformation plan (July 2015–June 2020) mentioned disability as a means of measuring equitable access to quality health services. Reducing health disparities through improved access to care for under-served populations including PWDs is a top priority on the Country’s health sector transformation plan [6]. These show that the country is doing something to addressing the SRH needs of PWDs at policy or strategy level.

People’s perception on disability can have positive or negative impacts on life experiences and opportunities, including seeking, accessing and using maternal health services. Many studies findings showed that stereotypical views and misconceptions regarding the sexual and reproductive lives of PWDs impede access to SRH services [7–10]. Assumptions like PWDS are not sexually active; are asexual, uninterested in sex, or unable to take part in sexual activity, and unable to control their sexual drives are widespread. The study done in three African countries namely Ghana, Uganda, and Zambia finds that women with disabilities (WWDs) are perceived to be HIV-free and having sex with a woman or girl with disabilities can cure HIV infection [7].

Ahumuza et al. finds an inherent societal misperception among interviewed people with disabilities in Uganda that PWDs do not need SRH services and information [8]. As a result, the healthcare delivery system has turned away PWDs from seeking and accessing SRH services. The research reports of the disability rights international and Colectivo Chuhcan’s from Mexico demonstrates that 69% of the women interviewed believed that WWDs cannot financially support child rearing and over 60% believed that WWDs should undergo medical tests before considering pregnancy to prevent her from passing on her disability [9].

These misconceptions and myths conceal real sexual relations and practices of people living with disabilities and enforce the idea that people with disabilities are incapable of starting sexual and marital relations; likely a reason for excluding intentionally or unintentionally them from normal sexual lives which are an integral part of human reproduction and pleasure. These misconceptions violate the SRH rights of PWDs and it exposes them to sexual violence, inadequate sexual and reproductive health care and HIV/AIDS [10, 11].

As far as our review of evidences, very little are known about the level of knowledge and use of contraceptive methods among blind and deaf women in Ethiopia. The findings of this research will assist decision makers, program designers and managers to develop sensory disability inclusive family planning services or programs so as to improve reproductive health status of women with sensory disabilities in Ethiopia and elsewhere. It will also play a significant role in mainstreaming these services in order to previously endorsed and upcoming reproductive health policies and strategies. Above all, it can also function as a footstep for further research.

## Study objectives and aims

The aim of this study was to determine the knowledge and practice level on modern contraceptive methods among blind and deaf women about in Addis Ababa City, Ethiopia. Specifically, the study aimed first to assess the sexual health characteristics of blind and deaf women, second to determine the knowledge level of blind and deaf women about modern contraceptive methods and last but not least to assess the practice of using modern contraceptive methods among blind and deaf women.

## Methods

### Study setting and period

We have conducted the study in Addis Ababa, the capital city of Ethiopia. Administratively, the City has ten sub-cities, which are the second administrative units next to city administration. There were no registered recent statistics showing the number of PWDs in Addis Ababa, and the available data are outdated. Based on the data obtained from Addis Ababa City Administration Health Bureau, as of June 2015, there were 86 governmental health centers and 13 hospitals in this City. In addition, there were more than 36 private hospitals and 700 low to higher-level private health clinics. The first phase, quantitative survey, was done from August 2016 to March 2017, and the second phase, qualitative study, was conducted in April 2017.

### Study design

A descriptive cross-sectional study design was employed using an explanatory sequential mixed method approach.

### Study population

The study populations for this study are women of reproductive age (15–49 years old) who are blind, or having severe blindness and woman who are deaf or having profound hearing loss from both ears and those who stayed in all 10 sub-cities of Addis Ababa for at least 1 year before the date of survey.

### Samples and sampling procedures

The quantitative sample size was calculated using a single population proportion formula, and proposed a total of 330 women (165 deaf and 165 blind) to be a sample. The respondents for the quantitative study were selected and approached using the Respondent Driven Sampling (RDS) technique (see Additional file 1). The qualitative study involved 29 key informants; 8 from disabled persons’ associations or organizations, 3 from relevant government sector bureaus and 19 from health facilities. In addition, 10 public health facilities in Addis Ababa City (one per sub-city) were observed regarding their accessibility for sensory disabled clients. We selected these participants using purposive sampling technique and recruitment of participants ceased when descriptive saturation was achieved.

### Data collection tools and methods

The quantitative data were collected by using a pre-tested semi-structured questionnaire which contained four sections; socio-demographic characteristics, reproductive and sexual health history, problems and risk factors, awareness and knowledge about family planning and practice of using family planning services (see Additional file 2). It was collected through face-to-face interview and facilitated by experts in special need education and had adequate prior experience in collecting data for similar surveys. One of them was a professional sign language interpreter. We have gathered the qualitative information using in-depth interviews (IDIs) with key informants and facility direct observation.

The questionnaire and other data collection tools were pre-tested during the preparatory work on 5% of the total required sample outside Addis Ababa City. Accordingly, we ensured the clarity, wording, logical sequence and skip patterns of the questions.

### Variables measurement

In this study, the outcome variables are knowledge level and practice of using contraceptive methods. The respondents’ comprehensive knowledge on contraceptive methods was measured based on the correct responses given to the eight knowledge tracing variables. As a result, comprehensive knowledge was measured if the score was more than 5 out of the 8 FP knowledge related questions and if the score was 4, it was labeled as fair knowledge and if it was 3 or less it was considered as not knowledgeable.

The practice of using contraceptive methods was assessed by asking the respondent whether she had ever the history of using any of the modern contraceptive methods for the sake of preventing pregnancy and the response was dichotomous in the form of yes or no.

In this study, we measured self-perception using 4 items with a yes or no options. These items are low self-confidence, dependency, inferiority and affect psychology. If the responses to these 4 questions/items was yes, then we considered the woman as having good self-perception.

### Data management and statistical analyses

The supervisors checked the completeness and quality of each filled questionnaire on daily bases during the field data collection. At the field level, the missing item/s have been rectified by revisiting that respondent through the data collector before leaving the village. Above all, the authors found two questionnaires which were incomplete, missed important variables and thrown out these questionnaires before data entry. We did the analysis using SPSS version 22 (See Additional file 3).

We undertook bivariate and multivariate logistic regression analysis to examine the net effects of a set of explanatory variables over the outcome variables. Statistical significance was set at a *p*-value of less than 0.05. The qualitative analysis was done using Open code software version 4.02 where themes were identified based on the objectives of the study and triangulated with the quantitative findings.

### Ethical clearance

We obtained the ethical approval certificate for the study from the Research Ethics Committee of the Department of Health Studies, University of South Africa (REC-012714-039). We also got a research permit from the Health Bureau of City Administration of Addis Ababa to facilitate the conduct of research activities in the community. All participants provided written informed consent. This study used the parental/guardian informed consent form and the assent form for under 18 year respondents. The interviewers given utmost respect throughout the whole process and maintained the privacy and confidentiality of survey respondents.

## Results

A total of 328 women (165 deaf women and 163 blind women) were interviewed, yielding a response rate of 99.4%. Since two of the questionnaires were thrown out before data entry because of the incompleteness of important variables, the analysis was done on 326 cases (164 deaf and 162 blind women).

### Socio-demographic characteristics of respondents

The mean age of the respondents was 28.57 years with a standard deviation of 7 years. By marital status at the time of survey, more than half (54.3%) were single. The minimum age at first marriage reported was 8 years and marriage before celebrating 18th birthday, child marriage, was 27.9%. About one to five (20.6%) of the respondents were illiterate meaning can’t read and write. By ethnic composition, 44.8% of the respondents belong to the Amhara followed by Oromo 22.7%. The dominant religion of the study population was Orthodox Christian (65.6%). By occupation, 36% of the respondents were doing their own small business, 11.7% were beggar and 17% were unemployed or didn’t have any work (Table 1). Pertaining to the residence (sub-city) distribution of respondents at the time of survey, nearly one-third (31.6%) of the respondents were living in Gulele, 49(15.0%) in Yeka, 35(10.7%) in Arada, an equal proportion (each 8.0%) in Bole and Addis Ketema, 25(.7%) in Kolfe Keraniyo, 23(7.1%) in Lideta, 14(4.3%) in Nifas-Silk Lafto and 12(3.7%) in Akaki kality of sub-city of Addis Ababa (Table 1).

**Table 1:** The distribution of selected socio-demographic Characteristics of respondents, July 2016–April 2017. Addis Ababa

Characteristics	Blind (%)	Deaf(%)	Total frequency (%)
Age group			
< 19 years	20 (12.3)	4 (2.4)	24 (7.4)
[20–29 years]	76 (46.9)	104 (63.4)	180 (55.2)
[30–39 years]	49 (30.2)	54 (32.9)	103 (31.6)
[40–48 years]	17 (10.5)	2 (1.2)	19 (5.8)
Current Marital status			
Married	39 (24.1)	52 (31.7)	91 (27.9)
Single	79 (48.7)	98 (59.7)	177 (54.3)
Divorced	31 (19.1)	12 (7.3)	43 (13.2)
Widowed	13 (8.0)	2 (1.2)	15 (4.6)
Educational status			
Illiterate (can’t read and write)	54 (33.3)	13 (7.9)	67 (20.6)
Primary school (Grade 1–8)	29 (17.9)	50 (30.5)	79 (24.2)
Secondary school (Grade 9–12)	33 (22.2)	34 (20.7)	67 (20.6)
College certificate/Diploma & above	46 (28.4)	67 (40.8)	113 (34.7)
Ethnicity			
Amhara	102 (63.0)	44 (26.8)	146 (44.8)
Tigrea	8 (5.0)	31 (19.0)	39 (12.0)
Oromo	25 (15.4)	49 (29.8)	74 (22.7)
SNNP^a^	26 (16.0)	40 (24.3)	66 (20.2)
Afar	1 (0.6)	0 (0.0)	1 (0.3)
Religion			
Orthodox	127 (78.4)	87 (53.0)	214 (65.6)
Islam	15 (9.2)	3219.5)	47 (14.4)
Protestant	20 (12.3)	42 (25.6)	62 (19.0)
Catholic	0 (0.0)	3 (1.8)	3 (0.9)
Occupation			
Employee^b^	29 (18.0)	27 (16.4)	56 (17.1)
Own business^c^	65 (40.1)	52 (31.7)	117 (35.9)
Student	20 (12.3)	37 (22.5)	57 (17.5)
Begging	31 (19.1)	7 (4.2)	38 (11.7)
Not engaged	17 (10.5)	41 (25.0)	58 (17.8)
Housing tenure			
Own house	18 (11.1)	65 (37.8)	83 (25.5)
Rented house	124 (76.5)	99 (60.3)	223 (68.4)
Institution based	18 (11.1)	0 (0.0)	18 (5.5)
Street based	2 (1.2)	0 (0.0)	2 (0.6)
Total	162	164	326
Age at first marriage (*n* = 140)			
< 18 years	31 (41.3)	8 (12.3)	39 (28)
18–22 years	25 (33.3)	28 (43.0)	53 (38)
23–27 years	14 (18.6)	17 (26.1)	31 (22)
28–33 years	5 (6.6)	12 (18.4)	17 (12)
Total	75	65	140
Currently living with (*n* = 308)			
Husband / children	72 (50.0)	59 (36.0)	131 (42.5)
Family members	22 (15.3)	66 (40.2)	88 (28.6)
Alone	19 (13.2)	6 (3.6)	25 (8.1)
Relatives	10 (7.0)	31 (18.9)	41 (7.5)
Friends / peers	21 (14.5)	2 (1.2)	23 (7.5)
Total	144	164	308

^a^Include Gurage, Wolaita, Gamgofa, Sidamo, Hadiya, and Kembata ethic groups

^b^ Include government, private company or NGO employees

^c^ Low earning jobs include selling lottery, coffee-tea, cultural clothes & arts, soft, mobile card, and others

### Sexual and reproductive health characteristics of respondents

Ninety percent of the study subjects remembered their age of menarche. The reported median age at menarche was 15 years for blind and 13 years for deaf women, and this has shown statistical significance with the age at menarche (*P* < 0.001). Earlier menarche is typical of deaf girls, and late menarche is the feature of blind girls. The proportion of respondents who reported ever having had sex was 65.3%. There is a statistically significant result between type of disability and sexual activeness (*P* < 0.001) in which blind women are more likely sexually active compared to deaf women (78.4% versus 52.4%). About 133 (62.4%) of the sexually women have had sexual intercourse in the last 12 months prior to the survey, yet 80(37.6%) have not had in the specified time.

The mean age at first sexual intercourse was 19.0 years with a standard deviation of 4.4 years and ranged between 9 and 32 years. More than two third, 145 (68.0%) of sexually active respondents have had at least one pregnancy (65.4% for blind and 72.1% for dear women).

Pertaining to risky sexual behaviors, 102 (48.0%) of sexually active respondents had only one while 111 (52.0%) had two or more sexual partner/s. The study also revealed that 42 (19.7%) of the sexually active respondents have had sexual intercourse with a non-regular unknown partner for the sake of getting financial or material benefits. Nearly one-quarter (24.4%) of sexually active women had ever used condom. Of whom only half (50.0%) of them reported proper and consistent use of condom. Surprisingly, three-quarter (75.6%) had never used condom. According to this study finding, the practice of sex after using khat or alcohol was reported by almost one-third (34.3%) of sexually active respondents (Table 2).

**Table 2:** Percentage distribution of sexual and reproductive health characteristics of respondents, July 2016 – April 2017. Addis Ababa

Characteristics	Blind (%)	Deaf (%)	Total frequency (%)
1. knows age of menarche?			
Yes	138 (85.2)	155 (94.5)	293 (90.0)
No	24 (14.8)	9 (5.5)	33 (10.0)
2. Age group at menarche			
10- -13 years	34 (24.6)	127 (82.0)	161 (55.0)
14–19 years	104 (75.4)	28 (18.0)	132 (45.0)
3. Median age at menarche	15 years	13 years	13 years
4. Ever had sex			
Yes	127 (78.4)	86 (52.4)	213 (65.3)
No	35 (21.6)	78 (47.6)	113 (34.7)
Total (1,2 &4)	162	164	326
5. Had sex in the last 12 months			
Yes	69 (54.3)	86 (52.4)	133 (62.4)
No	58 (45.7)	78 (47.6)	80 (37.6)
6. Mean age at first sexual act	18.3 + 4.2 year	20.1 + 4.6 year	19.0 + 4.4 year
7. Ever used condom consistently and properly			
Yes	36 (28.3)	16 (18.6)	52 (24.4)
No	91 (71.7)	70 (81.4)	161 (75.6)
8. History of pregnancy			
Yes	83 (65.4) 44	62 (72.1) 24	145 (68.1)
No	(34.6)	(27.9)	68 (31.9)
9. No of lifetime sexual partner			
One	69 (54.3)	33 (38.4)	102 (47.8)
Two and above	58 (45.7)	53 (61.2)	111 (52.2)
10. Ever practiced sex with unknown partner/s			
Yes	18 (14.2)	24 (27.9)	42 (19.7)
No	109 (85.8)	62 (72.5)	171 (80.3)
11. Practice of unprotected sex			
Yes	91 (71.6)	70 (81.4)	161 (75.6)
No	36 (28.4)	16 ((18.6)	52 (24.4)
12. Ever practiced sex after use of substances or drugs			
Yes	52 (40.9)	21 (24.4)	73((34.3)
No	75 (59.1)	65 (75.6)	140 (65.7)
Total (5–12)	127	86	213

### Prevalence of sexual and reproductive health-related problems

The prevalence of unwanted pregnancy was 67.0% among the survey respondents. The number of unwanted pregnancy ranged from one to four and 25 (36.0%) of them have faced unwanted pregnancy more than one times in their lifetime. Even though the prevalence of unwanted pregnancy was higher among blind women than deaf women (71.1% versus 61.3%), the association was not statistically significant (*p* = 0.215). The prevalence of abortion means terminating pregnancy before the age of 28 weeks of gestation was 44%. The mean number of abortion was 1.27 and the maximum number of abortion reported was four. Among women who had history of abortion, induced and spontaneous abortion were reported by 57 (89.0%) and 7(11.0%) of them respectively.

Moreover, 18.7% of survey respondents reported that they have complained at least one of symptoms of sexually transmitted infections (STIs) such as unusual or odd-smelling vaginal discharge, unusual vaginal bleeding, lower abdominal pain, rash over the trunk, hands or feet, itching, ulcer and painful or burning urination. Experience of at least one of these symptoms of STIs among blind women (25.3%) was almost twice as compared to deaf women (12.2%) and this difference was statistically significant (*p* = 0.02) (Table 3).

**Table 3:** Prevalence distribution of sexual and reproductive health-related problems among respondents by type of sensory disability, July 2016 – April 2017. Addis Ababa

Sexual and reproductive health-related problems	Blind women (%)	Deaf women (%)	Total (%)
Encountered unwanted pregnancy			
Yes	59 (71.2)	38 (61.3)	97 (67)
No	24 (28.8)	24 (38.7)	48 (33)
Total	83	62	
Ever had abortion			
Yes	37 (44.6)	27 (43.5)	64 (44.0)
No	46 (55.4)	35 (56.5)	81 (56.0)
Total	83	62	145
Type of abortion			
Induced	32 (86.5)	25 (92.6)	57 (89.0)
Spontaneous	5 (13.5)	2 (7.4)	7 (11.0)
Total	37	27	64
Experienced at least one of the symptoms of STIs			
Yes	41 (25.3)	20 (12.2)	61 (18.7)
No	121 (74.7)	144 (87.8)	265((81.3)
Total	162	164	326

### Awareness and knowledge about family planning methods

Accordingly, 317 (97.2%) of the study respondents had heard about FP methods. Regarding method specific awareness, the same number of women (316, 97.0%) had heard about oral contraceptive pills and injectable (Depo-Provera), 308 (94.5%) about implants, 311 (95.4%) about male condom, 282 (86.5%) about IUCD, 199 (61.0%) about emergency oral contraceptive, 117 (36.0%) about female condom, 236(72.4%) about breast feeding, 206(63.2%) about calendar, 127 (39.0%) about female sterilization, 107 (32.8%) about male sterilization, 97 (29.8%) about withdrawal method.

When responded to each method specific knowledge question, out of those who had heard about pills, 98 (31.0%) of them were not sure or did not know whether oral pills should be taken daily,10 (3.2%) were not sure or did not know whether injectable should be taken every 3 month, 84(27.3%) were not sure or didn’t know whether Implants can prevent pregnancy up to 5 years, 139 (49.3%) were not sure whether IUCD can prevent pregnancy up to 12 years, 52(16.7%) were not sure whether one male condom can’t be used more than once, 91(45.7%) were not sure whether emergency pills must be taken within 72 h after unprotected sex, 68(28.8%) were not sure whether breast feeding can prevent pregnancy up to 6 months after delivery, and 125 (60.7%) were not sure whether day 9–19 of the menstrual cycle are unsafe period (Table 4).

**Table 4:** Awareness and knowledge level of respondents on family planning methods. July 2016–April 2017. Addis Ababa, Ethiopia

Awareness about family planning methods	Responses	Frequency (%)
1. Heard about FP service	Yes	317 (97.2)
	No	9 (2.8)
2. Heard about Oral contraceptive pills	Yes	316 (97.0)
	No	10 (3.0)
3. Heard about Injectable (Depo-Provera	Yes	316 (97.0)
	No	10 (3.0)
4. Heard about Implants	Yes	308 (94.5)
	No	18 (5.5)
5. Heard about IUCD	Yes	282 (86.5)
	No	44 (13.5)
6. Heard about Male Condom	Yes	311 (95.4)
	No	15 (4.6)
7. Head about lactational amenorrhea method (breast feeding)	Yes	236 (72.4)
	No	90 (27.6)
8. Heard about calendar method	Yes	206 (63.2)
	No	120 (36.8)
9. Heard about female sterilization	Yes	127 (39.0)
	No	199 (61.0)
10. Heard about male sterilization	Yes	107 (32.8)
	No	219 (67.2)
11. Heard about withdrawal method	Yes	97 (29.8)
	No	229 (70.2)
Total		326
Knowledge indicators		
1. Oral pills should be taken daily (*n* = 316)	Yes	218 (69.0)
	No / Don’t know	98)31.0)
2. Injectable (Depo) should be taken every 3 months (*n* = 316)	Yes	306 (96.8)
	No / Don’t know	10 (3.2)
3. Implants can prevent pregnancy up to 5 years (*n* = 308)	Yes	224 (72.7)
	No / Don’t know	84 (27.3)
4. IUCD can prevent pregnancy up to 12 years (*n* = 282)	Yes	143 (50.7)
	No / Don’t know	139 (49.3)
5. One condom can’t be used more than once (*n* = 311)	Yes	259 (83.3)
	No / Don’t know	52 (16.7)
6. Emergency pills must be taken within 72 h after unprotected sex.	Yes	108 (54.3)
	No / Don’t know	91 (45.7)
7. Breast feeding can prevent pregnancy up to 6 months	Yes	168 (71.2)
	No / Don’t know	68 (28.8)
1.8. Day 9–19 are unsafe period of the menstrual cycle	Yes	81 (39.3)
	No / Don’t know	125 (60.7)

However, the analysis showed that comprehensive knowledge on FP was lower (32.5%). The level of knowledge on FP methods has shown significant association with the type of sensory impairment (*P* = 0.01). Deaf women were more likely to have comprehensive knowledge when compared with blind women.

### Source of information on family planning

The most commonly reported sources of information about family planning were friends/peers. The most commonly reported source of information about family planning methods was friends/peers, more than three-quarter (241, 76.0%) of them got information through their peers. Healthcare workers, television/radio and sexual partner were the source of information for 149 (47.0%), 135(42.6%), (80, 25.2%) of the surveyed women respectively.

### Practice of using contraceptive methods

Almost half of sexually active respondents ever used modern contraceptive method. Respondents were asked whether or not they were using contraceptive method at the time of interview, accordingly 31.1% of them were using. The proportion of blind women (66%) who reported ever use of FP service was much higher when compared to the proposition of deaf women (33.5%) and this difference was statistically significant (*P* < 0.001). The median age at start of using contraceptive methods was 21 years.

**Fig. 1:**
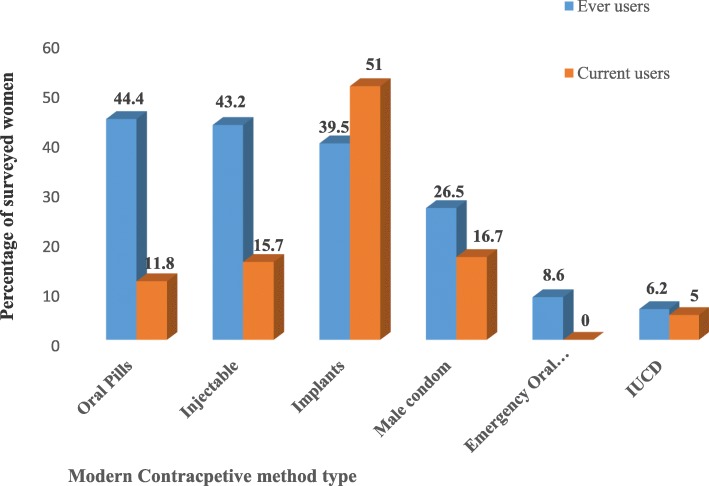
Ever and currently used modern contraceptive methods among survey respondents. July 2016 – April 2017. Addis Ababa, Ethiopia

Short-acting methods were the common methods used by ever users whereas implants were the most commonly used (51%) contraceptive method among current users. The share of IUCD was only 5.0%, short acting methods was 44.2% (Pills, 11.8%; Injectable, 15.7%, and male condom, 16.7%) among current users (Fig. 1).

Nearly one-third (32.4%) of current FP users used contraceptive method to space their childbirth, 29 (28.4%) to limit childbirth, 13(12.7%) of them for the purpose of delaying the time of their first pregnancy. Almost one fourth of them used because of fear of forceful sexual intercourse or rape. Most of current users (78.4%) have got the contraceptive method from public health facilities, 15 (14.8%) from pharmacies or drug stores and the remaining (6.8%) from private clinics.

**Fig. 2:**
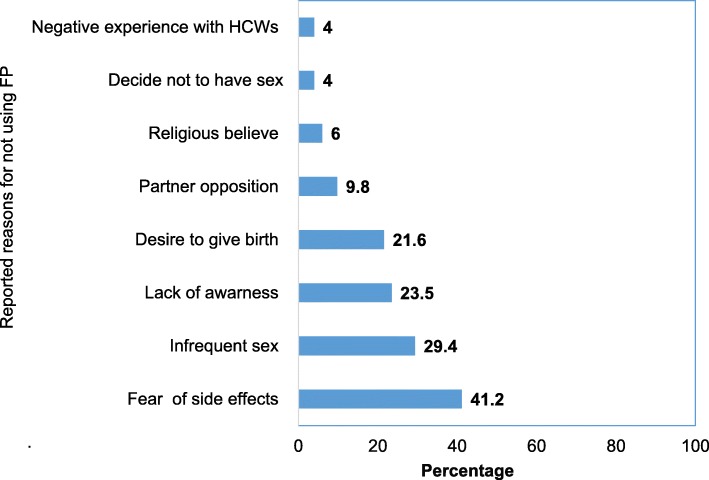
Percentage distribution of reported reasons for not using FP services among sexually active surveyed women. July 2016–April 2017. Addis Ababa, Ethiopia

Those sexually active respondents who had history of using contraceptive methods, yet discontinued at the time of survey (*n* = 51) were asked about their main reason, accordingly the common cited reasons were fear of side effects (41.2%), followed by having infrequent sex (29.4%), lack of awareness (23.5%) and desire to be pregnant and give birth (21.6%) (Fig. 2).

Multivariate logistic analysis revealed that women with higher age, in marital union, having good knowledge on FP methods, blind women and having good self-perception were more likely to use any modern method of contraception than the younger women (15–24 years), unmarried women, having poor knowledge, deaf and women having low self-perception (Table 4). Women in the age group of 35–48 years were three times (AOR = 3, 95% CI: 1.48–5.98) and those in the age group 25–34 years were 4.4 times (AOR = 4.37, 95% CI: 1.90, 10.04) more likely to use FP service compared to age groups of 15–24 years. Currently, married women were five times (AOR = 5.11, 95% CI: 2.48, 10.54) more likely to have history of using FP service as compared to not currently married women.

Women who perceived positive family members’ attitude were less likely (AOR = 0.48, 95% CI: 0.26, 0.88) to have FP service use when compared to women who perceived negative attitude of family members. Women having comprehensive or fair knowledge on FP methods and blind women were three times (AOR = 2.82, 95% CI: 1.47, 5.40) and six times (AOR = 6.40, 95% CI: 3.40, 12.01) more likely to use FP service as compared to women who do not have comprehensive FP knowledge and deaf women respectively. Moreover, women with good self-perception were less likely (AOR = 0.52, 95% CI: 0.28, 0.98) to have history of FP service use as compared to women with low self-perception. Blind women were 6.4 times more likely (AOR = 6.4,95% CI:3.40,12.01) to use family planning services when compared with deaf women (Table 5).

**Table 5:** Multivariable logistic regression analyses of selected factors affecting use of contraceptive methods among respondents. July 2106–April 2017. Addis Ababa, Ethiopia

Factors	Used contraceptive method	Crude OR (95%CI)	Adjusted OR (95%CI)	*P*-Value
	Yes (%)			
Age (in Year)				
15–24 year	27 (16.7)	1.00	1.00	
25–34 year	75 (46.3)	3.88 (2.23, 6.75)	2.98 (1.48, 5.98)	0.002
35–48 year	60 (37.0)	7.2 (3.80, 13.63)	4.37 (1.90, 10.04)	0.001
Marital Status				
In marital union	69 (42.6)	4.79 (2.77, 8.27)	5.11 (2.48, 10.54)	0.000
Not in marital union	93 (57.4)	1.00	1.00	
Type of sensory impairment				
Blind	107 (66.0)	3.86 (2.45, 6.10)	6.40 (3.40, 12.01)	0.000
Deaf	55 (34.0)	1.00	1.00	
Self-perception				
Good	90 (55.5)	0.40 (0.25, 0.65)	0.52 (0.28, 0.98)	0.043
Low	72 (44.5)	1.00	1.00	
Knowledge on FP methods				
Comprehensive/fair knowledge	125 (77.0)	2.08 (1.27, 3.39	2.82 (1.47, 5.40)	0.002
No knowledge	37 (23.0)	1.00	1.00	
Family attitude (*n*=				
Positive	90 (55.5)	0.47 (0.29, 0.75)	0.48 (0.26, 0.88)	0.018
Negative	72 (44.5)	1.00	1.00	

The qualitative results of this study identified various barriers and concerns which might contribute to the low level of knowledge and practice of using contraceptive methods among blind and deaf women. It was revealed that numerous misunderstandings and myths were pervasive, which disregard the sexuality and reproductive concerns, rights and aspirations of impaired women/girl’s in the community. The frequently coded responses were asexual consideration of WWDs followed by their incapability of practicing and leading romantic sexual life and of providing the necessary care for their newborn. The key informants pointed out that the members of the community consider impaired women as a burden who need care and they are unable to provide care for their children and husband.

*“There are also people in our community who assume that disabled peoples are asexual; do not have sexual feeling.”* (Key informant from the Ministry of Health).

**“.**. **.** *For example, how can a blind woman provide care for her children and fulfill the needs of husband on top of her impairment?” (Social worker from WWDs’ Association***).**

One of the common contributing factor for low comprehensive knowledge on modern contraceptive methods was lack of appropriate information communication means and modes which target persons with sensory impairments. Almost all of the key-informants shared this concern.

*“In the absence of accessible IEC materials targeting PWDs and sign language interpretation, how can sensory impaired people get and understand the information and have good level of knowledge? The situation is worst among those who are illiterate; who can’t read and access written sources of information? If we look at the mass media, they don’t convey sign language supported messages or programs on SRH topics.”* (Healthcare service provider from one of the health facility).

Some key informants also posed questions in relation to information access;

*“How can blind people hear information if it is not transferred using audio and how can deaf people access information where sign language is very limited in our setup? If we look at the mass media, they don’t convey sign language supported messages or programs. If present, they are rare and depend on special occasions. In such condition how can sensory impaired people get and understand the information and have the required knowledge? The situation is worst among those who are illiterate*.” (HIV counselor and sign language interpreter).

The observation findings also revealed that there were no any written, visual or audio materials at assessed health facilities for persons with sensory impairments.

Qualitative study participants have mentioned the common sexual and reproductive health-related problems women with sensory disabilities have been facing. This SRH problems were unwanted pregnancy, abortion, and sexual violence.

*“Out of the four disabled women I had encountered during my service experience, three of them become pregnant outside marriage and the pregnancies were unwanted. Two of them become pregnant as a result of rape by her relative.”* (Healthcare service provider).

It was also reported that service providers have had very little training in relation to disability and limited access to the resources that would enable them to provide a disability inclusive SRH services.

*“I don’t know whether this health center has a staff who has sign language training or not. Personally, I do not have such type of training. So far, I didn’t hear such type of training.” (*Healthcare service provider from another health facility).

## Discussion

Our study showed that blind and deaf women are sexually active and sought SRH services which is in line with the results of many studies which confirmed the sexual activeness of women with various disabilities [11–15]. This implies that sensory impairment does not preclude the person from being sexually active and the potential demand of WWDs for SRH services. It also gives a clue for healthcare managers and decision makers to design a program which could address SRH services needs of these segments of the population. However, the qualitative findings revealed the presence of misconceptions and stereotypes in the community pertaining to the sexuality of WWDs. These assumptions and myths lead to seeing women with impairments as they are free from SRH risks like HIV infection and virgin. In additions, these may create misimpression in the community like sexuality is not on the radar of girls or mothers with impairments and that sexuality is not an appropriate topic of discussion for them.

The analysis showed that comprehensive knowledge on FP was generally low (32.5%). This is in agreement with the findings of the qualitative study conducted by FHI 360 [16]. This may be attributed to the communication barriers, lack of information, unavailability of sign language interpreters in health facilities and non-tailored media based dissemination of information. These were the possible reasons mentioned by the participants of the qualitative study.

The most commonly reported sources of information about family planning were friends/peers followed by healthcare workers and television/radio. This finding is in line with a study conducted by Rugoho & Maphosa in Chitungwiza town of Zimbabwe [17].

Almost half of sexually active respondents ever used modern contraceptive method and 31.1% of the respondents were using at the time of survey. This finding is encouraging when compared to the results of the study done in Bahr Dar City in which 37.5% of them ever used modern contraceptive method and 25.2% of them were using at the time of survey [18].

If FP utilization is low, on the other hand, the number of unwanted pregnancy and abortion is more likely to increase. Coming to the low uptake of modern contraceptive methods by blind and deaf women, different factors contribute their own share; the reported knowledge gaps regarding why, how, where and when to use contraceptive methods could result in low demand for contraceptive methods. Many studies have demonstrated that knowledge of contraceptive methods is the key factor in proper and effective use of the method. Being knowledgeable rectifies the rumors, misconceptions and fears result in favorable attitudes towards use [19–21]. These findings may signal the high-unmet need for contraception among sexually active WWDs. Moreover, the pervasive stereotypes against WWDs in matters related to sexuality, mainly the perception that they do not need contraception because they are asexual, contributes to low uptake of the FP service as revealed by the results of the qualitative study. The result is low when compared with the results of the 2016 Ethiopian Demographic survey where 50% of currently married women in Addis Ababa reported use of modern contraceptive methods [22].

This study demonstrated that short-acting methods were the most commonly used among ever users. This is similar to the findings reported by the Ethiopian health and demographic survey and other studies [18, 22]. Implants were the frequently used method among users at the time of the survey. This may imply the preference of non-user dependent methods by blind and deaf women and their intention to avoid repeated visit to health facilities by choosing long acting reversible contraceptive methods such as Implants, which can provide protection for an extended period. The findings of this study can be generalized to other similar study settings, samples, or populations, taking into account the relevant facts and internal validity of the study.

The findings of this study have policy relevant implications. To mention the main; it signals the importance of addressing equity in health care service provision and becoming inclusive of PWDs in the planning and implementation SRH services at all the level of the health care system. This may require specific reforms and actions across the health system mainly in areas of service delivery, infrastructure, human resources, and information systems. The findings also inform SRH program managers and policy makers to develop service delivery guideline so as to standardize the provision of sensory disability friendly SRH services at health facilities in Ethiopia.

## Limitations of the study

This study may have its own limitations in that some medical terms were difficult for the sign language expert to translate exactly using sign language when interviewing deaf women. The cross-sectional nature of the quantitative study does not allow causal inferences about the association between FP service utilization and those independent factors. The study was also very limited to the capital city where most of the residents have better education, health facilities are highly widespread and findings might not reflect the situation to the rural part of the country.

## Conclusions and recommendations

Women with sensory disability are sexually active; however, most of them were not using modern contraceptive methods at the time of survey. Thus, women with sensory disability are at risk of unwanted pregnancy and abortion. The government need to address the attitudinal, social, and physical barriers women with sensory disabilities are facing while seeking, accessing to and using family planning services. Health facilities and concerned organizations should create awareness among women with sensory disabilities and the local community using appropriate communication channels and medias regarding their sexual and reproductive health rights, family planning options, and where and when to get these services. This action helps to break the negative social image and enable the community members and FP service providers to be friendly to women with sensory disabilities.

## Supplementary information

**Additional file 1.** Details of sampling technique employed.

**Additional file 2.** Survey instrument.

**Additional file 3.** Dataset.

## Data Availability

The datasets used and/or analyzed during the current study are available from the corresponding author on reasonable request. The survey instruments used to generate the data in this study and the details of the sampling technique employed are included as additional files to this manuscript.

## Abbreviations

IDI: In-depth interviews
IUCD: Intra Uterine Contraceptive Device
PWDs: Person with Disabilities
RDS: Respondent Driven Sampling
SPSS: Statistical Package for Social Sciences
SRH: Sexual and Reproductive Health
UNCRPD: UN Convention on the Rights of Persons with Disabilities
WHO: World Health Organization
