# Supplementary material for: Modern contraceptive methods knowledge and practice among blind and deaf women in Ethiopia. A cross-sectional survey
Source: BMC Womens Health. 2019 Nov 29;19:151. doi: 10.1186/s12905-019-0850-y (PMC6884853; doi:10.1186/s12905-019-0850-y)
Supplement: Supplementary file 1 — Additional file 1. Details of sampling technique employed. [file 12905_2019_850_MOESM1_ESM.doc]

#### Details of the sampling technique employed

The researcher has used the Respondent Driven Sampling (RDS) technique to select and contact survey respondents because of two basic reasons. The first reason was difficulty of getting and constructing complete and comprehensive sampling frame, which include women fulfilling the inclusion criteria. It was also a challenge to know their exact size and boundaries. The second reason was the hidden population feature of these populations, namely, women with sensory disabilities. PwDs are considered hidden populations and difficult to study. Most of these individuals do not have a fixed address. Thus, it is difficult to reach them through household surveys or venue-based samplings.

Respondents driven sampling (RDS) is a new form of chain-referral sampling (CRS) method that relies on a structural systems of recruitment procedures and financial incentives to encourage recruitment and participation (Iguchi, Ober, Berry, et al. 2009:7).

RDS was practiced in recent studies for sampling hidden population mainly HIV/AIDS, female sex workers, gays and bisexual men, and drug users (Ludlam, Saxton, Dickson, & Adams 2015:2; Lansky et al. 2012:78). However, researchers criticise that other chain-referral sampling methods such as snowball, targeted and time-space for sampling hidden populations are not effective in making statistical inferences. However, RDS makes possible probabilistic sampling when surveying hidden target population (Iguchi et al. 2009:10). These authors also noted that RDS addresses the limitation of snowball sampling by overcoming the bias of the initial seeds, the final sample become completely independent of the initial seeds.

Qualitative analysis of peer recruitment pressures in RDS indicates that peer recruitment practices in RDS generally pose minimal study-related risks (Mosher, Moorthi, & Weeks 2015:9). Therefore, RDS uses an indirect method to make estimates from the sample to the population of interest; it makes estimates from the social network to the population. By doing this, RDS avoids many of the well-known problems with estimation from chain-referral samples. Accordingly, I strongly argue that women with sensory disabilities fulfil the aforementioned assumptions of RDS. Hence, RDS is the appropriate sampling procedure, which enabled me to produce unbiased estimates through reaching all members of the target population. I described the steps followed in the next sections.

##### Step 1. Selection and Recruitment of initial seeds

Owing to the heterogeneity of the target population under study and intention of including survey respondents from different occupations. I have separated the target population into two broad groups based on the type of sensory disability, namely, totally blind and totally deaf or profoundly hearing impaired women. Then, three categories were formed under each group taking into account the various distinct groups and social networks in the target populations. The first category represented the social network from members of the national association of the blind and deaf, the second category comprised the network from those who have been leading their job or life on the street, and the third group included the network from those who were visiting religious places (Church/Mosque).

This enabled me to reach and involve those who do not have the chance to the public venues and are not members of disability association and it helped me to consider different groups that exist in the target population. Thus, it enhanced the validity and reliability of the samples. Exerts or social workers from each of the national association of the blind, deaf, and centre for the blind actively participated during the selection of the initial seeds. As illustrated in the figure below, after listing three potential cases for each category, we have randomly selected one of these cases and included it as an initial seed. By doing these, all segments of women with sensory disabilities were effectively represented and included with non-zero probability.

Women with sensory disabilities

Totally blind women

**C1**

Totally deaf or profoundly hearing impaired women

**C1**

**C2**

PS1

PS2

PS3

**C2**

**C3**

**C3**

PS1

PS2

PS3

PS1

PS2

PS3

PS1

PS2

PS3

PS1

PS2

PS3

PS1

PS2

PS3

SSC

SSC

SSC

SSC

SSC

SSC

**SRS**

Key: Category 1- Members of the association

Category 2- Working or leave on the street

Category 3- Student (regular or Non-regular)

PS- Potential seed

SSC= Selected seed from each category

SRS- Simple random sampling

**Selection processes of the initial seeds**

##### Step 2. Designing survey participants’ recruitment coupon

At the same time, I have given emphasis for the prevention of repeated participation by recording unique identifier for each participant during the recruitment and interview process. The interviewers analysed, compared the information with the participant’s database, and checked if the participant had been previously interviewed.

The recruitment coupon has three parts. The first part highlights the objectives of the research and provide information, the second part deals about recruiters and the third part focuses on the recruits. One coupon was used only once and coming with this coupon was must to include the woman. The card displayed below was the recruitment coupon.

| Overview of the Research | Recruiter’s part | Recruits’ part |
| --- | --- | --- |
| Information sheet  ------------------------------  ---------------------  Objectives of the research  -----------------------------  ------------------------  Researcher: ___________  Sign: _____________  Mobile: +251-942202698 | Date: ________________  **Women-code**  Appointment date: _________ | Date: ___________  **Women-code**  Appointment date: ________  Valid only for 3 weeks |
|  | Interviewer: _________________  Sign_________________  Mobile: ________________  N.B: Keep this coupon and bring it to interview in order to receive incentives | |

##### Step 3. Selecting and arranging interview places and venues

Interviews were carried out in private, safe, comfortable, and convenient places. Taking into account the dispersed samples within wide coverage area of the study site, Addis Ababa City, the interviews were conducted at six locations, which are centrally located and would reduce transportation-related barriers. In addition, potential participants from one side of the city may find it difficult to get interview site on the other side of the city. In addition, a phone call was made to arrange an interview appointment in advance. If in case the recruit did not come on the scheduled interview date, repeat calls were made to get the recruit.

##### Step 4. Decision on the Incentive system

Recruiters received their monetary incentive in two phases. The first phase payment was effected after completing their interview and the second phase payment was made after screening the waves and ensuring that the wave met the study inclusion criteria. This payment was given to the recruiter through one of the wave. I have also noted the problems associated with too high amount of incentives, about $2 for a single wave.

##### Step 5. Recruitment of the first and subsequent waves

The first waves consists of initial seed’s recruits and those recruits then found peers who made the second waves of the chain and so on. I have provided 1-3 unique recruitment coupon, which was valid for only three weeks period. When approaching the wave respondents, interviewers did not ask them about their peer who recruited them, verified their eligibility, explained the consent of the study, and kindly invited them to participate in the study.

The waves composed the chain and the chains finally resulted in the sample. When adequate chain was not formed from the initial seeds, new seeds were recruited from the under sample group and began a new RDS waves. Finally, the seed chains ranged from 30 to 85 and interviewers stopped the RDS after getting the required samples from each group (blind and deaf). I have also verified that the final samples were completely independent of the initial seeds.
