# Supplementary material for: Modern contraceptive methods knowledge and practice among blind and deaf women in Ethiopia. A cross-sectional survey
Source: BMC Womens Health. 2019 Nov 29;19:151. doi: 10.1186/s12905-019-0850-y (PMC6884853; doi:10.1186/s12905-019-0850-y)
Supplement: Supplementary file 2 — Additional file 2. Survey instrument. [file 12905_2019_850_MOESM2_ESM.doc]

**EGLISH VERSION QUESTIONNAIRE**

Questionnaire Identification Number: |___|___|___|

Interview start: ____/___/_______

Interview ended: ____/_____/_____

**Address of respondents**

Sub city: __________________

Woreda/Kebele: ______________________

Village: _________________________

**Section 1: Background Characteristics of the respondent**

| Q.No | Questions | Response and coding categories | Skip |
| --- | --- | --- | --- |
| 101 | How old were you at your last birthday? | Age in completed years   |  |  | | --- | --- | |  |
| 102 | Current Marital status? | 1. Married 2. Single  3. Divorced 4. Widowed | **2->104** |
| 103 | Age at first marriage | ___________________ |  |
| 104 | Religion? | 1.Orthodox 2.Islam  3.Protestant 4.Catholic  88. Others (specify:_____________________) |  |
| 105 | Ethnicity? | 1.Amhara 2.Tigray  3. Oromo 4. Gurage  5. Wolaita 6. Gamogofa  7. Afar 8. Sidama  88.Others (specify:____________________) |  |
| 106 | What is your level of education? | 1. No schooling – can’t read/write  2. No formal school, but Only read/write  3. _________________ completed grade  4. TVET/College Diploma  5. University Degree and above |  |
| 107 | Where do you live currently?  (The usual place) | 1. My own home 2. Rented house 3. Institution based 4. Street based   88. Others (specify:______________________) |  |
| 108 | With whom do you live currently?  Who do you share your house with? | 1. Husband/Boyfriend/ Sons/daughters 2. Father/Mother/brother/sister 3. Alone 4. Relatives (Uncle/Aunt/…..) 5. Friends/peers (Female) 6. Others (specify:______________________) |  |
| 109 | Number of family size including you? | __________________ |  |
| 110 | What is your current occupation? | 1.Gov’t employee  2. Private company employee  3. Non- gov’t organization employee  4. My own business  5. Student  6. Beggar  88. Other (Specify: ------------------------------------) |  |

**Section 2. Reproductive Health History, Problems and Risk Factors**

| Q.No | Questions | Response and coding categories | | | Skip |
| --- | --- | --- | --- | --- | --- |
| 201 | Age at menarche? | 1. ----------- years  2. Don’t remember  3. I don’t want to respond to this | | |  |
| 202 | Do you have or ever had boyfriend/partner  For those who are not currently married  Compare with Q.N 102 | 1. Yes , I have 2. Yes , I have had 3. No | | | **3->205** |
| 203 | Is your boyfriend/partner also have impairment? | 1. Yes - ________________type 2. No | | |  |
| 204 | Do you always accept and execute what your boyfriend/partner has said/requested? | 1. Yes 2. No 3. If not convinced I do not accept | | |  |
| 205 | Have you ever had sexual intercourse? penis-in-vagina sex | 1. Yes  2. No | | | **2->section 3** |
| 206 | At what age did you first have sexual intercourse? | 1. ______ years  2. Don't remember | | |  |
| 208 | How many sexual partners do you have so far? | 1. __________________ man  2. I don’t want to respond  99. Don’t know exactly | | |  |
| 207 | Have you had sexual intercourse in the last 12 months? | 1. Yes  2. No | | |  |
| 208 | Have you ever used condom properly? | 1. Yes  2. No | | |  |
| 209 | How frequently did you use condom? | 1. Always(consistently)  2. Most of the time  3. Rarely /sometimes | | |  |
| 210 | Have you used condom the last time you had sexual intercourse? | 1. Yes 2. No | | |  |
| 211 | If you have never used condom, what was your reason?  Read each option |  | Yes | No |  |
|  |  | 1. Difficulty in using it properly |  |  |  |
|  |  | 1. Partner opposition |  |  |  |
|  |  | 1. I trust my partner |  |  |  |
|  |  | 1. It reduce my sexual pleasure |  |  |  |
|  |  | 88. Others (specify) _____ |  |  |  |
| 212 | Have you ever practiced sex with non-regular partner for the sake of exchanging benefits/gifts | 1. Yes 2. No | | |  |
| 213 | Have you ever practiced sex under the influence of Khat/alcohol, shisha, hashish | 1. Yes 2. No | | |  |
| 214 | Have your partner ever practiced sex with you under the influence of Khat/alcohol, shisha, hashish | 1. Yes 2. No | | |  |
| 215 | Have you ever been pregnant?  Including abortion and still birth | 1. Yes 2. No | | |  |
| 216 | How many times have you been pregnant? | ------------- time | | |  |
| 217 | How old were you when you first became pregnant? | 1.------------ years  2 .Don’t remember | | |  |
| 218 | How many times you encountered unwanted pregnant? | 1. No history of unwanted pregnancy 2. Yes ______________ times | | |  |
| 219 | Have you ever given birth?  Excluding abortion | 1. Yes 2. No | | |  |
| 220 | How old were you when you gave your first birth? | 1.__________years  2.Don’t remember | | |  |
| 221 | How many births you have given? | ____________ births | | |  |
| 222 | How many life children of your own you have? | _______________ | | |  |
| 223 | Have you ever had abortion? (Termination of pregnancy before 28 weeks of gestation) | 1. No  2. Yes  99. I don’t want to respond | | |  |
| 224 | Number of abortion? | __________ times | | |  |
| 225 | What was the nature of abortion? | 1. Induced  2. Spontaneous | | |  |
| 226 | Have you ever been raped (forced sexual intercourse including unwilling sex)?  Compare with Q.N 205 | 1. Yes  2. No | | |  |
| 227 | Who was the perpetrator? | 1. Boy friend  2. Strange person 3.Teacher  4.Close relative  5.Neigbour  88.Others(specify) ________________ | | |  |
| 228 | Your main reason for not practicing sex?  More than one answer is possible | 1. Fear of influencing my plan and goal 2. No one asked me for marriage 3. Thinking that the exact time will come 4. Fear of the consequences 5. Fear of the upset from my families 6. Not acceptable in my community   88.Others(specify) ________________ | | |  |

**Section 3: Awareness and knowledge based questions about family planning methods**

| Q.No | Questions | Response and coding categories | | | | | Skip |
| --- | --- | --- | --- | --- | --- | --- | --- |
| 301 | Do you ever heard or have information about family planning methods? | 1. No  2. Yes | | | | |  |
| 302 | What type of family planning methods do you know?  (Multiple answer is possible) |  | | Yes | No | |  |
|  |  | 1. Oral contraceptive pills | |  |  | |  |
|  |  | 2. Injectable | |  |  | |  |
|  |  | 3. Implants | |  |  | |  |
|  |  | 4. Male Condom | |  |  | |  |
|  |  | 5. Female Condom | |  |  | |  |
|  |  | 6. IUCD | |  |  | |  |
|  |  | 7. Female sterilization | |  |  | |  |
|  |  | 8. Emergency contraceptive(pill) | |  |  | |  |
|  |  | 9. Male sterilization | |  |  | |  |
|  |  | 10. Calendar/Rhythm method | |  |  | |  |
|  |  | 11. Withdrawal | |  |  | |  |
|  |  | 12.Exclusive breastfeeding(LAM) | |  |  | |  |
|  |  | 88. Other (specify) _________ | | | | |  |
| 303 | Your knowledge on the various contraceptive methods you have heard  Read each options |  | Yes | | | No | Don’t know |
|  |  | 1. Oral pills should be taken daily |  | | |  |  |
|  |  | 1. Implants can prevent pregnancy upto 5 years |  | | |  |  |
|  |  | 1. Injectable(Depo) should be taken every 3 month |  | | |  |  |
|  |  | 1. IUCD can prevent pregnancy upto 12 years |  | | |  |  |
|  |  | 1. One condom can’t be used more than once |  | | |  |  |
|  |  | 1. Emergency pills must be taken within 72 hours after unprotected sex. |  | | |  |  |
|  |  | 1. Breast feeding can prevent pregnancy upto 6 months |  | | |  |  |
| 304 | Which is your major source of information about the family planning method you heard?  (Multiple answer is possible) | 1. TV/Radio/FM program  2. Health professionals  3. School Teachers  4. Leaflets/Newspapers/magazine  5. Partner/Boy friend  6. Friends/peers  7. Parents (family members)  88. Others (specify: -----------------------------) | | | | |  |

**Section 4. Questions concerning family planning service utilization**

| Q.No | | Questions | Response and coding categories | | | Skip |
| --- | --- | --- | --- | --- | --- | --- |
| **Family Planning Services** | | |  | | |  |
| 401 | Have you ever used any family planning methods to delay or avoid getting pregnant? | | 1. No  2.Yes | | | **1-> Q 410** |
| 402 | Which modern family planning method you have used?  (multiple answers are possible) | | 1. Oral contraceptive pills  2. Injectable  3. Implanon  4. Jadelle  5. Male Condom  6. Female Condom  7. IUCD  8. Female sterilization  9. Male sterilization  10. Emergency contraceptives (Pills)  88. Other (specify) ---------------------------------- | | |  |
| 403 | At what age did you first start using modern method of contraception? | | Age |__|__|  99. Don’t remember | | |  |
| 404 | Are you currently using contraceptive method to delay or avoid getting pregnant? | | 1. Yes  2. No | | | **2-> Q 407** |
| 405 | Which family planning method you are currently using?  Condom is circled if she use it for the purpose of avoiding pregnancy , not to protect from HIV | | 1. Oral contraceptive pills   2. Injectable  3. Implanon  4. Jadelle  5. Male Condom  6. Female condom  7. IUCD  8. Female sterilization  9. Male sterilization  10. Natural FP methods  88. Other (specify:_____________________) | | |  |
| 406 | For what purpose you are currently using a contraceptive method? | | 1. Birth spacing  2. Limiting birth  3. Want to delay pregnancy  4. Fear of forced sex or rape  88. Other (specify: ___________________) | | |  |
| 407 | The main reason why you are not using contraceptive currently?  Read each option | |  | Yes | No |  |
|  |  |  | 1. Desire to have more children |  |  |  |
|  |  |  | 1. Fear of side effects |  |  |  |
|  |  |  | 1. Decided not to perform sex |  |  |  |
|  |  |  | 1. I have infrequent sex |  |  |  |
|  |  |  | 1. Husband/partner opposed |  |  |  |
|  |  |  | 1. Religious Prohibition |  |  |  |
|  |  |  | 1. Preferred method not available |  |  |  |
|  |  |  | 1. Lack of knowledge. |  |  |  |
|  |  |  | 1. Healthcare providers’ negative attitudes |  |  |  |
|  |  |  | 1. The health institutions are too far |  |  |  |
|  |  |  | 1. Not supported by my family |  |  |  |
|  |  |  | 1. Difficult to reach at health facility |  |  |  |
|  |  |  | 1. Financial constraint |  |  |  |
|  |  |  | 88. Others (specify:__________________) | | |  |
| 408 | Do you intend to use family planning in the future to delay or avoid pregnancy? | | 1. Yes  2. No | | |  |

That is the end of our questionnaire. Thank you very much for taking time to answer these questions.
